# Bilateral orbital myeloid sarcoma preceding acute myeloid leukemia in an adult: a case report and review of the literature

**DOI:** 10.1186/s13256-015-0778-2

**Published:** 2016-02-04

**Authors:** Jesus Vera-Aguilera, Osama Mukarram, Prathibha Nutalapati, Mary Mok, Anushi Bulumulle, Carlos Vera-Aguilera

**Affiliations:** 1 Internal Medicine Department, Texas Tech University Health Sciences Center at the Permian Basin, 800 W. 4th Street, Odessa, TX 79763 USA; 2 Departamento de Biología Celular y Tisular, Facultad de Medicina, Universidad Nacional Autónoma de México, México City, Mexico

**Keywords:** Acute myeloid leukemia, Cancer, Hematology, Myeloid sarcoma, Unusual presentation

## Abstract

**Background:**

Acute myeloid leukemia is typically a disease of the older population and presents mostly in the fifth decade of life. Myeloid sarcoma is a rare initial presentation of acute myeloid leukemia. Previously it has only been documented in children and younger patients.

**Case Presentation:**

We present an unusual case of retro-orbital myeloid sarcoma as an initial presentation of acute myeloid leukemia in a 43-year-old Caucasian man, with rearrangement of chromosome 11q23 involving the *MLL* gene.

**Conclusions:**

We present an unusual case of retro-orbital myeloid sarcoma as an initial presentation of acute myeloid leukemia in a 43-year-old man, with rearrangement of chromosome 11q23 involving the MLL gene.

## Background

Acute myeloid leukemia (AML) is generally a disease of older population with an average age of presentation of 66 years. It is uncommon before the age of 45 [1]. Several disorders preceding the onset of AML have been reported. These include myelodysplastic syndrome (MDS), myeloid dysplasia, aleukemic leukemia cutis, hemophagocytic syndrome, and myeloid sarcoma (MS) [2–6]. MS may be the first manifestation of AML, precede it in presentation by months or years, or even present as the initial manifestation of the relapse of a disease in remission [7, 8]. It has been reported in 2–8 % of patients with AML either as a single or as a multifocal tumor. MS contains myeloblasts, with or without features of promyelocytic or neutrophilic maturation. Frequently, MS displays myelomonocytic or pure monoblastic morphologic features [8]. It may be present in variable locations, such as skin, bone, lymph nodes, and very rarely in the retro-orbital area [4, 7].

## Case presentation

A 43-year-old Caucasian man presented to our emergency room with frontal headache and blurred vision. He had a recent history of surgically excised malignant melanoma of the right cheek and right-sided Bells’ palsy treated with acyclovir and steroids. His vital signs on arrival were as follows: temperature 36.9 °C, blood pressure 129/79 mmHg, heart rate 68 beats per minute, respiratory rate 18 breaths per minute, and arterial oxygen saturation of 93 % on room air. A neurological examination revealed weakness in adduction of his left eye with complete ptosis (likely due to left third nerve palsy), ipsilateral conjunctival injection and dilated pupil, a depressed nasolabial fold on the left side, and drooping of the angle of his mouth to the left with drooling (representing left seventh nerve palsy). His deep tendon reflexes were decreased to 1+ in his upper and lower extremities along with generalized decreased sensation to pin prick and light touch. Motor strength was preserved in all his extremities. The results of the rest of a physical examination were within normal limits.

**Fig. 1:**
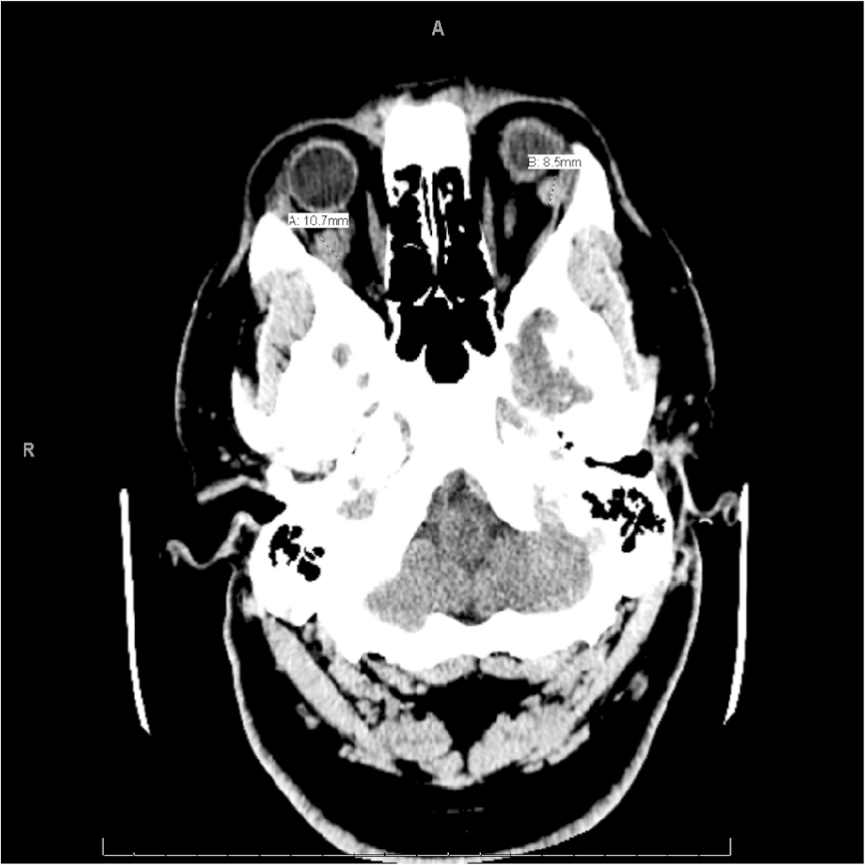
Computed tomography scan of the head showing broad-based multiple bilateral retro-orbital masses along the medial side of the lateral rectus muscle, the largest one measuring 10 mm

Blood work showed that our patient had a white blood cell (WBC) count of 5.6 × 10^3^ /μL, hemoglobin of 14.6 g/dL, and platelet count of 268 × 10^3^ /μL, all within normal limits. His serum chemistry and results of a chest X-ray were also normal. However, a computed tomography (CT) scan of his head (Fig. 1) showed broad-based multiple bilateral retro-orbital masses along the medial side of the lateral rectus muscle, the largest one measuring 10 mm. No abnormalities were observed on magnetic resonance angiography or CT angiography of his head. We started our patient on vancomycin, ceftriaxone, and acyclovir while awaiting lumbar puncture results. Infectious and autoimmune analysis, including human immunodeficiency virus, Lyme’s disease, cryptococcosis, coccidioidomycosis, West Nile virus, California encephalitis, St Louis encephalitis, eastern equine encephalitis, antinuclear antibodies and antineutrophil cytoplasmic antibodies, had negative results. A lumbar puncture showed clear fluid with glucose 108 mg/dL, total protein 95 mg/dL, 1 WBC/mm^3^, and 1 red blood cell/mm^3^. No malignant cells were seen in the cerebrospinal fluid.

Our patient complained of intractable headache and was started on intravenous methylprednisolone 1 g/day, which alleviated his symptoms. On day 8, a repeat CT scan of his head showed no changes compared to previous studies and our patient underwent a needle biopsy of the left orbital mass. The biopsy results were inconclusive because only adipose tissue could be sampled, without any evidence of malignant cells. A CT scan of his thorax and abdomen was done owing to the high suspicious of malignancy, and no signs of malignancy were appreciated. Our patient was transferred to a specialized cancer center for further evaluation, where he was treated with intravenous immunoglobulins and plasmapheresis for suspected atypical Guillain-Barré syndrome presentation.

**Fig. 2:**
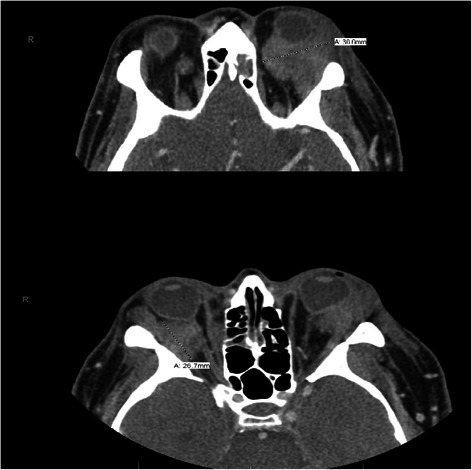
Computed tomography scan of the head showing increase in size of the orbital masses, measuring 3.7 cm on the left and 2.7 cm on the right, causing a mass effect on the optic nerves

Two months later, our patient’s symptoms persisted and he underwent another orbital biopsy that found atypical lymphocytes. These lymphocytes stained positive with Leder stain, indicating their origin to be myeloid. The next day our patient developed fever; a neurological examination showed no changes compared to his previous admission. A repeated complete blood count showed a WBC count of 1.1 × 10^3^/μL [neutrophils 15 %, absolute neutrophil count (ANC) of 0.17 × 10^3^/μL, monocytes 19.8 %, lymphocytes 63 %], hemoglobin 7.7 g/dL, and a platelet count of 66 × 10^3^/μL. Our patient was started empirically on vancomycin, piperacillin-tazobactam, and levofloxacin. A CT scan (Fig. 2) of his head showed an increase in size of the orbital masses, measuring 3.7 cm on the left and 2.7 cm on the right, causing a mass effect on his optic nerves. Blood cultures remained negative for viral infections. On hospital day 8, our patient’s neutropenia worsened, his complete blood count showed a WBC count of 0.78 × 10^3^/μL (neutrophils 9.4 % with ANC 0.07 × 10^3^/μL, monocytes 40 %, lymphocytes 50 %), hemoglobin 7.4 g/dL, and a platelet count of 25 × 10^3^/μL. A bone marrow biopsy was performed, which showed 79 % blast cells with almost no myeloid maturation present, consistent with AML. Flow-cytometry showed immune-reactivity for stem cell markers CD34 and CD117, a normal CD4 to CD8 ratio, and pan-T-cell antigens. A cytogenetic study using florescent *in situ* hybridization analysis revealed a rearrangement of chromosome 11q23 involving the *MLL* gene. The clinical course of this patient was complicated by *Clostridium difficile* colitis. It was decided to start the patient on induction chemotherapy once his infective status improves. In the meantime, a lower extremity ultrasound was done for his leg swelling and showed bilateral distal lower extremity deep venous thrombosis. Given our patient’s thrombocytopenia, an inferior vena cava filter was placed to prevent pulmonary embolism. On day 9, our patient developed ventricular fibrillation, and died after a cardiac arrest.

## Discussion

The National Cancer Institute estimated for 2015 a total of 20,830 new cases of AML and 10,460 deaths due to AML and associated complications in the USA [9]. MS represents a particular form of AML in which a tumor-like proliferation of blast cells occurs outside of the bone marrow [10]. Previously, soft tissue accumulations of leukemic cells were referred to as granulocytic sarcoma (GS) or chloroma. These are rare localized extramedullary tumor masses comprising myeloid precursor cells [11]. A total of 87 % of GS biopsies were found to be Leder stain-positive, indicating a myeloid origin of the cells, as seen in our patient [12].

While several variants of AML have few or no cells of granulocytic lineage, the broader term “myeloid sarcoma” is currently preferred [13]. MSs are most common in certain subtypes of AML, particularly M5a (monoblastic), M5b (monocytic), M4 (myelomonocytic), and M2 (myeloblastic with maturation) [14]. Orbital GSs have mostly been reported in African, Asian, South Pacific, and Latin American children. They are extremely rare in adult patients with AML [15]. MS may develop de novo or concurrently with AML, myelo-proliferative neoplasm (MPN), or MDS.

Cytogenetically, MS has been associated with a variety of chromosomal abnormalities, including *MLL* gene rearrangement and t(8;21) translocation. It usually corresponds to AML with a French-American-British (FAB) classification of M4 or M5 [8, 16–19]. The presence of retro-orbital masses preceding AML is rare. To the best of our knowledge, since 1993 a total of 11 cases of GS preceding AML in adults have been reported [10, 20–27], most of them presenting with the cytogenic t(8:21) translocation and a fair prognosis. In the present report, we describe a very aggressive case of AML positive for CD34 and CD117 and a rearrangement of chromosome 11q23 involving the *MLL* gene that resulted in death. These genetic abnormalities comprise a category of recurring genetic abnormalities in the World Health Organization classification. In a case series, the incidence of 11q23/*MLL* rearrangement was found to be 2.8 % of almost 2,000 patients and was associated with poor outcome [28]. Interestingly, CD117, a transmembrane protein receptor encoded by the c-kit proto-oncogene, initially viewed as a primitive myeloid marker, was present in our patient. CD117 has been identified in all FAB subtypes of AML and approximately 4 % and has been associated with poor outcomes [29].

Differential diagnoses in adults who present with similar symptoms are broad and require a high index of suspicion. Priego *et al*. recommend that the differential diagnosis should include inflammatory/metabolic disease (orbital inflammatory pseudo-tumor, thyroid orbitopathy, sarcoidosis) and neoplasm (lacrimal tumors, lymphoma, and metastasis) [30]. However, clinical behavior and response to therapy do not seem to be influenced by age; sex; anatomic site; de novo presentation or clinical history related to AML, MDS, or MPN; histotype; phenotype; or cytogenetic findings [4, 8].

## Conclusion

MS, or GS, is an uncommon malignant neoplasm associated with AML. The presence of retro-orbital masses preceding AML is rare and few cases have been reported, most of them presenting with the cytogenic t(8:21) translocation and a fair prognosis. The present case is noteworthy because we describe a very aggressive case of AML with rearrangement of chromosome 11q23 involving the *MLL* gene that resulted in death. The differential diagnosis for patients of this age is broad and diagnosis can be challenging, therefore a multidisciplinary approach and an appropriate clinical examination and history, accompanied by a high index of suspicion, are needed for proper diagnosis and treatment.

## Consent

Written informed consent for publication could not be obtained despite all reasonable attempts at contacting our deceased patient's next of kin. Every effort has been made to protect the identity of our patient and there is no reason to believe that our patient would have objected to publication.
